# Does the opening of producer services promote the wage growth of the downstream manufacturing industry?—Empirical evidence from Chinese manufacturing listed companies

**DOI:** 10.1371/journal.pone.0293915

**Published:** 2024-04-18

**Authors:** Zhibin Zhang, Dian Wang

**Affiliations:** 1 Business School of Hunan University of Science and Technology, Xiangtan, Hunan Province, China; 2 Research Center for Regional High-Quality Development, Hunan University of Science and Technology, Xiangtan, Hunan Province, China; Sichuan Agricultural University, CHINA

## Abstract

Based on the vertical connection between upstream and downstream industries, a unique theoretical model is constructed to analyse the impact mechanism of the opening of producer services on downstream manufacturing wage growth. The empirical tests are carried out using the data of China’s manufacturing listed companies from 1999 to 2020. Our findings indicate that the opening of producer services has an inverted-U-shaped impact on downstream manufacturing wage growth, and the average level of the opening of producer services in the sample period is lower than the corresponding threshold. Overall, it is in the stage of promoting the wage growth of the downstream manufacturing industry. The opening of producer services mainly affects the wage growth of the downstream manufacturing industry through two channels: labour productivity and labour income share. The results of heterogeneity analysis show that the wages of capital and technology-intensive and low-competitive manufacturing industries are relatively strongly promoted by the opening of producer services. Therefore, promoting the orderly opening of producer services and strengthening the technological links between industries will help promote the wage growth of downstream manufacturing industries.

## 1 Introduction

The new round of the industrial revolution has greatly promoted the innovation of manufacturing production methods and service business models and has become an important force to reshape the global competitive pattern [1]. The competition for the dominance of international rule-making has also begun to shift from the manufacturing sector to the service sector [2], and the proportion of productive services trade characterized by knowledge and technology intensiveness has been rising year by year and has become a new trade growth point. Therefore, the academic community has begun to study the impact of the opening of service trade.

Many scholars believe that productive services are embedded in manufacturing production activities in an all-round way through the input‒output relationship, which effectively improves manufacturing production efficiency [3], driving technological progress and innovation [4], and promoting it to climb to the middle and high end of the global value chain [5]. It is bound to have an important impact on the labour supply and demand structure and even the income distribution pattern of the manufacturing industry [6]. However, few scholars have explored the specific mechanism between the development of the service industry and the manufacturing industry’s wage level from the industrial correlation perspective and the interindustry spillover effect. Liang and Cong (2019), from the perspective of industrial linkages and interindustry spillover effects, using spatial econometric analysis methods, found that the diversification and agglomeration of the service industry promoted the wage level of the manufacturing industry, while the specialization and agglomeration of the service industry had no significant effect on the wage level of the manufacturing industry [7]. Chen and Wei (2019) believe that the improvement of the degree of service-oriented manufacturing in the region will significantly increase the nominal wage income of manufacturing workers, and the development of the service industry and the improvement of the service industry level can effectively alleviate the problem of the excessive income gap [8]. From the perspective of the macro policy of producer services opening to the outside world, there is still much room for research on the theoretical model and interaction mechanism of its impact on the wage growth of downstream manufacturing industries.

**Fig 1:**
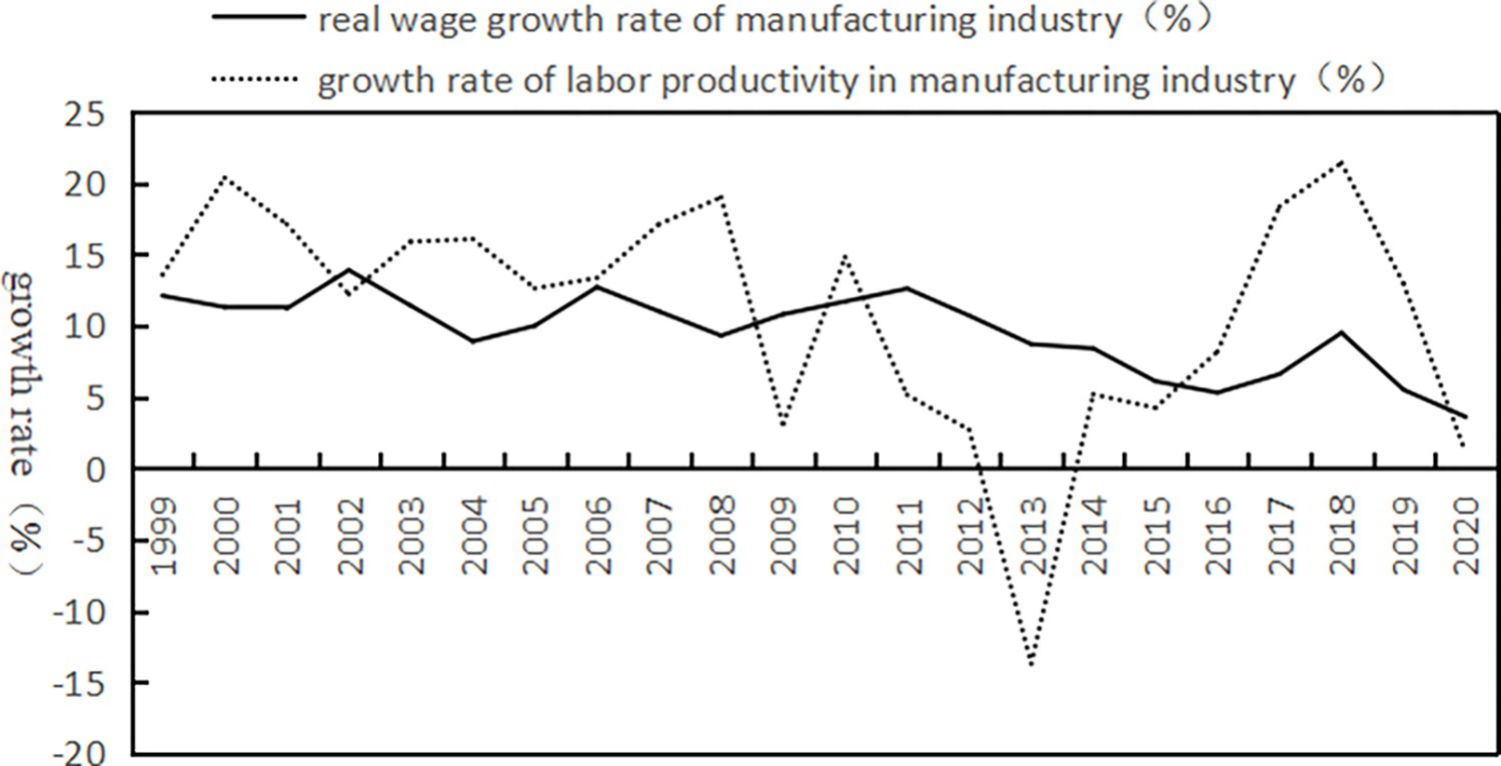
Trends of the real wage growth rate and labour productivity growth rate in China’s manufacturing industry (1999–2020). Data source: Calculated and sorted according to the China Statistical Yearbook and China Labour Statistical Yearbook over the years.

The reasons why we choose China’s service industry and manufacturing industry for research are as follows: on the one hand, since China formulated the "13th Five-Year Plan" strategy, which detailed the grand blueprint for economic and social development from 2016 to 2020, China has introduced a series of measures to expand the opening of foreign investment in the service sector, such as establishing and improving the negative list management system for cross-border trade in services and continuing to relax market access in the service sector. [9]. China’s position as the second-largest service outsourcing recipient and the second-largest cloud service provider has been increasingly consolidated. Thus, this paper chooses China as the research object. On the other hand, since the end of the 20th century, while continuing to maintain high-speed economic growth, China has also accumulated a series of obvious structural problems, which are mainly manifested in the widening income gap [10], imbalance in demand structure [11], and prominent structural contradictions on the supply side [12]. As shown in Fig 1, from 1999 to 2008, the average growth rate of labour productivity in China’s manufacturing industry was 15.73%, 4.6 percentage points higher than the average growth rate of real wages in the manufacturing industry. The sharp increase in labour remuneration in China’s manufacturing industry is the result of labour productivity. After the international financial crisis in 2008, due to the adverse impact of the international economic environment, the productivity growth of China’s manufacturing industry showed a downwards trend until 2013. From 2008 to 2020, the real wages in the manufacturing industry increased by 8.29% on average, and it can be seen that it has been difficult for labour productivity to continuously promote the improvement of labour remuneration [13]. Therefore, how to use the opening of producer services to transform and upgrade, eliminate the shackles of "low-end locking" of the manufacturing industry, and drive the sustainable growth of real wages is a theoretical problem that urgently needs to be solved in the new stage of China’s opening. Clarifying the relationship between the openness of producer services and the wage growth of the manufacturing industry will help to promote the international division of labour and industrial transformation of China’s manufacturing industry, drive the sustainable growth of labour productivity and labour income share of the manufacturing industry, drive the rise of the wage level of the manufacturing industry, further release the domestic consumption potential, and coordinate the tension between fairness and efficiency in the initial distribution. In summary, the research issues of this paper mainly include the following four aspects:

Q1: Will the openness of producer services affect the growth of real wages in downstream manufacturing?
Q2: What is the specific mechanism by which the openness of producer services affects the wage growth of the manufacturing industry?
Q3: Is there heterogeneity in the effect of different producer services opening on the wage growth of downstream manufacturing industries?
Q4: How can the openness of producer services be used to promote the transformation and upgrading of the manufacturing industry and the rise of global value chain status?

Compared with the existing studies, the innovations of this paper are as follows: (i) On the research theme, based on the perspective of vertical correlation between industries, we explore the impact of producer services opening on manufacturing wage growth. (ii) In terms of theoretical mechanisms, producer services are integrated into the wage growth model of manufacturing enterprises with production input factors, and a theoretical framework is established in which the openness of producer services affects the wage growth of manufacturing industries through labour productivity and labour income share. (iii) In the empirical analysis, the paper constructs an indicator of producer services opening that can better avoid endogeneity and discusses the difference in producer services opening to wage growth in different manufacturing industries.

This paper is arranged as follows. The next section combs the literature related to the openness of the service industry and the wage level of the manufacturing industry. After that, the development hypothesis section deduces and analyses the theoretical model of the main research hypothesis of the article. Then, the empirical model and variable definition are mainly proposed in the methods section. Second, the research hypothesis proposed above is empirically tested. In the final discussion section, in addition to discussing the results, it also points out the conclusions of this paper, proposes policy recommendations, and discusses the limitations of this research and future research directions.

## 2 Literature review

The literature related to this paper can be summarized in the following three aspects. One is about the impact of opening on income distribution. Some scholars believe that expanding the opening will aggravate wage inequality [14]. The expansion of opening will promote the market share to flow to enterprises with higher productivity and increase the wages of skilled and unskilled workers, but the income improvement effect on skilled workers is obviously stronger than that on unskilled workers [15]. Other studies have found that opening not only increases the income of low-skilled workers but also reduces their income level, leading to greater income inequality [16]. Some scholars hold the opposite view, believing that opening can reduce the overall income gap. Opening up to the outside world promotes the rapid growth of labour-intensive industries through the introduction of foreign capital, expands the demand for medium- and low-end labour, and then improves its relative wage level, accelerates the flow of labour, drives the rapid increase of the income of high-income rural strata, and reduces the wage gap of different skilled labour [17]. However, after technological progress is included in the analysis, it is found that there is heterogeneity in the relationship between opening and income inequality at different stages of technological development, presenting a nonlinear inverse U-shaped relationship [18]. Jalil (2012) also believes that there may be a curve relationship between openness and inequality, that openness variables may replace economic growth under the framework of the Kuznets curve and that income inequality may decline as openness reaches a turning point. China’s empirical research also shows that the promotion effect of opening on China’s labour income presents a nonlinear trend of first increasing and then decreasing [19, 20].

The second is to explore the impact of the opening of producer services on the development of the downstream manufacturing industry from the perspective of industrial correlation. Under the background of globalization, the opening of producer services can directly reduce the production cost of the manufacturing industry through the "competition effect" and "division effect” or improve production efficiency and relatively reduce the unit cost of products. On the one hand, the producer services industry promotes market competition by "introducing" foreign advanced service elements, reduces the relatively high price of domestic scarce service elements, gives manufacturing enterprises the opportunity to purchase high-quality intermediate service inputs at relatively low prices, greatly reduces transaction costs, produces direct cost reduction effects [21], and promotes the improvement of enterprise production efficiency [3]. The opening of producer services can also force Chinese manufacturing enterprises to strengthen R&D innovation and technological progress through the "competition avoidance" effect, which is conducive to improving product quality and brand value [22]. On the other hand, the opening of producer services can interact with domestic manufacturing enterprises through FDI, bring new technology, capital and management expertise, and improve the ability and level of domestic manufacturing enterprises to participate in the international division of labour [23]. The opening of producer services can effectively reduce information friction among enterprises and help enterprises carry out cooperative production, thereby reducing configuration costs, improving the quality of export products, and strengthening the positive impact of production segmentation on the quality of export products [24]. In conclusion, the "promotion effect" of the opening of producer services mainly plays a role through the cost savings of manufacturing enterprises triggered by the competition mechanism and the optimization of resource allocation triggered by the division of labour mechanism [25].

Some scholars also discussed the relationship between the openness of the service industry and the wage gap in the service industry. From the perspective of gender differences, skills-biased technological progress triggered by service elements will increase labour input with good interpersonal skills [26]; that is, the opening of the service industry has increased the demand for cognitive skill-intensive female labour [27], improved their labour remuneration, and reduced the input of sports skill-intensive conventional manual and conventional cognitive male labour [28]. From the perspective of industry differences, Zhong and Liu (2013) found that FDI in the service industry has raised the wage level of productive service industries and lowered the wage level of traditional consumer service industries, thus changing the relative wage level of productive service industries and consumer service industries [29].

The above documents mostly explore the heterogeneous impact on the wage income of different groups from the perspective of the opening policy or explore its impact on the wage gap within the industry from the perspective of the opening of foreign investment in the industry. They seldom consider the vertical relationship between upstream and downstream industries and the spillover effect between industries, especially going deep into the industry to capture the factors that affect the growth of labour income in the manufacturing industry. On the other hand, although many scholars have investigated the specific mechanism of the opening of producer services to drive the transformation and upgrading of the downstream manufacturing industry from the perspective of industry linkages, such as production efficiency, technological progress, or innovation, the relationship with wage growth, which represents the "residents’ happiness" index, has not been studied [30]. Based on the vertical relationship between input and output, this paper constructs a theoretical model and empirically analyses the impact mechanism of producer services opening on downstream manufacturing wage growth, which enriches the research in related fields to a certain extent.

## 3 Hypothesis development

Based on the wage growth model, this paper takes producer services as the intermediate input of manufacturing enterprises into the production function, explains the specific path of the opening of producer services affecting the wage growth of the manufacturing industry from the perspectives of labour productivity and labour income share, and proposes the research hypothesis of this paper combined with theoretical analysis.

### 3.1 Wage formation mechanism

Referring to Liu (2016), the production function model is set in Cobb Douglas form here to build a basic framework that includes the relationship between the growth rate of wages, labour income share, and labour productivity [31]. Namely:

$$Y=F(L,K)=AL^{\alpha }K^{\beta }$$ (1)

where *Y* is the output, *A* is the technical level, *L* is the labour input, *K* is the capital input, and *α* and *β* are the output elasticity of *L* and *K*, respectively. Then, the profit obtained by the manufacturer can be expressed as:

$$\begin{array}{l} \pi =PY-(WL+RK)\\ \;=PAF(K,L)-(WL+RK) \end{array}$$

where *π* is the manufacturer’s profit, *W* is the nominal wage, and *P* and *R* represent the product and capital prices, respectively. To maximize profits, the first derivative of the profit function must be equal to 0, that is:

$$\begin{array}{l} \frac{\partial \pi }{\partial L}=P\times \frac{\partial F(K,L)}{\partial L}-W\\ \;=P\times \frac{\partial F(K,L)}{\partial L}-W\\ \;=0 \end{array}$$

After sorting: $w=\frac{W}{P}=MP_{L}$

$$w=MP_{L}=A\alpha L^{\alpha -1}K^{\beta }=\alpha \frac{Y}{L}P=\alpha y$$ (2)

where *w* is the real wage level, *MP*_*L*_ is the labour marginal product, and *y* is the labour productivity. In addition, according to Formula (3), *α* can also be expressed as the share of labour income.


$$labor\;income\;share=\frac{WL}{PY}=\frac{w}{y}=\alpha$$ (3)

Then, we obtain the growth rate relationship among the real wage level, labour productivity and labour income share:

$$\frac{\Delta w(t)}{w(t)}=\frac{\Delta \alpha }{\alpha }+\frac{\Delta y(t)}{y(t)}$$ (4)

According to Formula (4), the growth rate of real wages is equal to the sum of the growth rate of labour productivity and the growth rate of labour income share [32]. Next, we will analyse the specific path by which the opening of producer services affects the wage growth of downstream manufacturing industries from two channels: labour productivity and labour income share.

### 3.2 Preferences and demands

It is assumed that the utility function obtained by consumers in consumption is in the form of a constant substitution elasticity (CES) utility function:

U=[∫i∈Ωqi(σ−1)σdi]σ(σ−1) (5)

where *i* represents the product type, σ is the product substitution elasticity, and σ>0. q_i_ is the consumer’s demand for product *i*, and Ω is the feasible product set.

According to the principle of utility maximization, the specific expression of the demand function *q*_*i*_ can be deduced:

$$q_{i}=R\frac{p_{i}^{-\sigma }}{G^{1-\sigma }}$$ (6)

G(τi)=[∫i∈Dpi1−σdi+∫i∈F(τipi*)1−σdi]1(1−σ) (7)

Among them, *τ*_*i*_ is the acquisition cost of foreign products or services, which is manifested in tariffs or restrictions on trade in services. *D* and *F* are the domestic and foreign product sets, respectively, *p*_*i*_ and *p*_i_^*^ are the prices of domestic and foreign products and services, respectively, R represents the total social income, and *G* represents the total price index of products.

According to Formula (7), $\frac{\partial G(\tau_{i})}{\partial \tau_{i}}>0$, that is, a reduction in the acquisition cost of imported products or services will lead to a reduction in the price index. Assuming that the labour input quantity *l*_*i*_ of the manufacturing industry is not affected by the opening of the upstream producer service industry, the labour productivity (*y*_*i*_*/l*_*i*_) is derived from τ_i_, and the following is obtained:

∂(yili)∂τi=li−1R1σ(1−1σ)(qi−1σG1−1σ∂qi∂τi+qi1−1σG−1σ∂G∂τi) (8)

It can be seen from Formula (8) that the impact direction of the opening of producer services on labour productivity is determined by the size of σ. If 0<σ< 1, ∂G(yili)∂τi<0, that is, when the substitution of domestic products or services for foreign products or services is weak, the opening of producer services has a positive effect on the labour productivity of downstream manufacturing industries. In contrast, when σ> 1, ∂G(yili)∂τi>0, that is, when the substitution elasticity of domestic products or services to foreign products or services is greater than 1, the opening of producer services will inhibit the improvement of labour productivity of downstream manufacturing industries.

The opening of producer services can improve the labour productivity of the downstream manufacturing industry through the “labour division effect”, “technology spillover effect” and “resource allocation effect”. Specifically, under open conditions, a large number of high-end service products enter the local market, which can fully solve the problem of insufficient supply of service factors [33]. This encourages enterprises to outsource the service links in the production chain to a third party, which not only improves the level of production specialization but also helps enterprises focus on the production and R&D links of products with competitive advantages and high added value and indirectly reduces production costs while improving production efficiency [34]. Moreover, the opening of the productive service industry has attracted foreign enterprises to enter the local market, brought advanced technology and management experience, promoted technological innovation and diffusion, promoted technological progress, increased the proportion of complex labour and the quality of labour, and further improved the labour production rate and management efficiency of the manufacturing industry [35]. The opening of producer services can also improve the degree of correlation between industries, optimize the allocation of factors among different industries, and improve the efficiency of resource allocation and labour production in the manufacturing industry.

On the other hand, the opening of producer services will also have an inhibitory effect on the improvement of labour productivity of downstream manufacturing industries through the “competitive effect”. “Introducing” a large number of foreign enterprises into the local market, intensifying competition in the domestic market and products [36, 37], forcing local enterprises to reduce their production scale [38], reducing their market share [39], and thus inhibiting the growth of labour productivity of domestic enterprises [40]. It should be noted that while expanding the opening of producer services promotes the rapid growth of exports, it may also lead to the low-quality “extensive trap” of domestic export trade [41]. Restricted by their own comparative advantages, developing countries are relatively low in the global value chain, vulnerable to the pressure of competition from the chain owners and the shackles of “low-end locking” [38]. It is difficult to obtain the corresponding added value, which has a negative impact on the improvement of the labour productivity of manufacturing enterprises and is not conducive to the growth of the wage level of the manufacturing industry.

### 3.3 Production department

Referring to the setting of the production function by Bentolila (2003) [42] and Prachi (2022) [43], it is assumed that the income distribution of each factor is variable, and the production function is set as CES:

$$Y_{it}={\left[(1-a){\left(B_{it}L_{it}\right)}^{\frac{\varepsilon -1}{\varepsilon }}+a{\left(A_{it}K_{it}\right)}^{\frac{\varepsilon -1}{\varepsilon }}\right]}^{\varepsilon -1}$$ (9)

where *K*_*it*_ represents the number of intermediate inputs of productive services consumed by manufacturing industry *i*. *a* and *(1-a)* represent the output elasticity of *K* and *L*, respectively. *ε* represents the substitution elasticity between elements. *B*_*it*_ and *A*_*it*_ indicate the efficiency of labour and service output, and this paper allows the existence of biased technological progress. The labour income share can be expressed as Formula (10):

$$S_{Li}=\frac{(1-a){\left(B_{it}L_{it}\right)}^{\frac{\varepsilon -1}{\varepsilon }}}{(1-a){\left(B_{it}L_{it}\right)}^{\frac{\varepsilon -1}{\varepsilon }}+a{\left(A_{it}K_{it}\right)}^{\frac{\varepsilon -1}{\varepsilon }}}$$ (10)

The input‒output ratio *k*_*it*_ of productive service elements can be expressed as:

$$k_{it}={\left(\frac{K_{it}^{\frac{\varepsilon -1}{\varepsilon }}}{(1-a){\left(B_{it}L_{it}\right)}^{\frac{\varepsilon -1}{\varepsilon }}+a{\left(A_{it}K_{it}\right)}^{\frac{\varepsilon -1}{\varepsilon }}}\right)}^{\frac{\varepsilon }{\varepsilon -1}}$$ (11)

Combining Eqs (10) and (11), we can obtain the expression (12) of labour income share:

$$S_{Li}=1-a{\left(A_{it}\;k_{it}\right)}^{\frac{\varepsilon -1}{\varepsilon }}$$ (12)

Expanding the opening of the productive service industry greatly improves the input quantity and quality of intermediate products of productive services in the production process [44]. To obtain the analytical expression, the input quantity *(K*_*it*_*)* of productive services is set as follows:

$$K_{it}=K_{it0}\;\tau_{i}^{-\lambda }$$ (13)

*K*_*it0*_ refers to the exogenous initial productive service input of the enterprise. 0< *λ*< 1 is the open knowledge and technology spillover coefficient of the producer service industry introduced for the amount of enterprise producer service input.


$$\frac{\partial S_{Li}}{\partial \tau_{i}}=-a\frac{\varepsilon -1}{\varepsilon }{\left(A_{it}\;k_{it}\right)}^{-\frac{1}{\varepsilon }}A_{it}\frac{\partial k_{it}}{\partial \tau }$$ (14)

Combining Formulas (11) and (13) to obtain $\frac{\partial k_{it}}{\partial \tau_{i}}<0$, the opening of producer services has increased the input of downstream manufacturing enterprises to producer services. Therefore, the final symbol of $\frac{\partial S_{Li}}{\partial \tau_{i}}$ depends on ε. If *ε* > 1, then $\frac{\partial S_{Li}}{\partial \tau_{i}}>0$; that is, when productive services and labour factors are mutually substituted, expanding the opening of productive services will reduce the labour income share of the manufacturing industry. When 0<*ε*<1, then $\frac{\partial S_{Li}}{\partial \tau_{i}}<0$; that is, when the productive service input and labour factors are complementary, expanding the opening of the productive service industry will increase the labour income share of the manufacturing industry.

The opening of producer services will have different effects on the labour income share of the manufacturing industry through a “direct effect” and “indirect effect”. On the one hand, from the perspective of input‒output, the opening of producer services will have a direct “ripple effect” on downstream manufacturing enterprises, drive the technological progress of the downstream manufacturing industry, strengthen the demand of downstream manufacturing enterprises for medium- and high-skilled workers, accelerate the allocation and optimization of human capital, improve the bargaining power of medium- and high-skilled workers, drive the growth of the wage level of the manufacturing industry, and increase the share of labour income.

On the other hand, the opening of producer services has an inhibitory effect on the growth of the labour income share of the manufacturing industry through an “indirect effect”. Taking advantage of their competitive advantages in the field of productive services, developed countries have directly infiltrated the manufacturing production process of other countries, especially developing countries, through the form of industrial chain owners, which has suppressed the cultivation of product R&D and innovation capabilities of manufacturing enterprises, resulting in the manufacturing industry falling into the shackles of “low-end locking” of the value chain and the reduction of the negotiation ability of labour factors [45]. At the same time, the participation of high-end service elements in the value-added benefit distribution mechanism of the domestic manufacturing industry may play a leading or controlling role in the value chain due to its strong productive service capacity and scarcity characteristics, resulting in the decline of the actual international division of labour [46], thus inhibiting the increase in the labour income share of the manufacturing industry.

Combined with the previous analysis, when the substitution of domestic products or services for foreign products or services is weak, expanding the openness of producer services will improve the labour productivity of the manufacturing industry through the "division effect", "resource allocation effect" and "technology spillover effect", which will positively promote the wage level of the manufacturing industry. However, with the continuous improvement of the level of opening to the outside world and the gradual shift of the economy to high-quality development, the "competitive effect" of the opening up of producer services through "introduction" is greater than the positive effect, which has a negative impact on the labour productivity of the manufacturing industry and inhibits the growth of the wage level of the manufacturing industry. When the inputs of producer services and labour are complementary, the openness of producer services improves the proportion of labour income through the "direct effect", and has a positive effect on the wage level of the manufacturing industry. As the economy changes from an "industrial economy" to a "service economy", there is little room for product quality differentiation, which cannot be translated into wage income. The labour income distribution effect of the opening of producer services will change from positive to negative, thus inhibiting the growth of manufacturing wages. Based on the above, this paper proposes the following three hypotheses.

*Hypothesis 1*: The opening of producer services has an inverted U-shaped nonlinear effect on the wages of downstream manufacturing industries, which increases first and then is suppressed.
*Hypothesis 2*: The opening of producer services can improve the labour productivity of the manufacturing industry through the labour division effect, technology spillover effect and resource allocation effect and drive an increase in the wage level. The opening of producer services will also inhibit the improvement in the labour productivity of the domestic manufacturing industry through the competitive effect and have a negative impact on the wage level of the manufacturing industry.
*Hypothesis 3*: The opening of producer services can increase the labour income share of the manufacturing industry through a “direct effect” and drive an increase in the wage level. The opening of producer services will also inhibit the increase in the labour income share of the domestic manufacturing industry through an “indirect effect” and have a negative impact on the wage level of the manufacturing industry.

## 4 Methods

### 4.1 Data description

The empirical study of this paper mainly involves four types of data: the microenterprise data mainly come from the CSMAR database of China. The data of China’s manufacturing listed companies from 1999 to 2020 were processed as follows: the data of ST, ST* and missing enterprises were excluded, and the samples with several enterprises in the industry less than 5 were deleted. A total of 418 manufacturing industry year regression sample observations were obtained. The input‒output coefficient of producer services to manufacturing comes from the input‒output table of 122 departments in 2002. The data on foreign capital participation in the service industry are from the catalogue of foreign investment industry guidance in 1997, 2002, 2004, 2007, 2011, 2015, and 2017. Table 1 shows the descriptive statistical results of each variable.

**Table 1:** Descriptive statistics.

	Variable interpretation	Mean	Standard deviation	Minimum	Maximum
**Wage**	Industry average wage level	2.490	0.727	1.052	5.575
**Msfdi**	Foreign investment opening index	0.902	0.207	0.535	1.311
**Capital**	Ratio of total fixed assets of the industry to total number of employees	2.420	0.708	1.044	8.755
**Size**	Logarithm of the total industry assets	7.427	1.386	3.651	10.589
**HHI**	Herfindahl index	0.100	0.064	0.014	0.3545
**Equity**	Average ratio of total industry liabilities to shareholders’ equity	0.794	5.859	0.043	5.484
**ROE**	Net profit as a percentage of average shareholders’ equity	0.089	0.029	0.028	0.305

### 4.2 Model setting

To test the nonlinear relationship between the opening of producer services and the wage growth of downstream manufacturing enterprises, an econometric Model (15) is established according to the theoretical analysis and research hypothesis:

$$Wage_{jt}=\eta_{0}+\eta_{1}Msfdi_{jt}+\eta_{2}Msfdi_{jt}^{2}+\varphi X_{jt}+\mu_{j}+\mu_{t}+\varepsilon_{jt}$$ (15)

The explained variable *Wage*_*jt*_ is expressed as the per capita real wage of manufacturing industry *j* in year *t*. The explanatory variable *Msfdi* is the degree of foreign capital regulation of the upstream producer services of manufacturing industry *j*. The larger *Msfdi is*, the higher the degree of foreign capital regulation of the upstream producer services of manufacturing industry *j*, and the lower the degree of openness. *X*_*jt*_ represents the control variable at the industry level. In addition, the model also controls the year and the fixed effect of the industry and controls the influence of the industry and the year and other related factors on the wage level of the manufacturing industry. *ε* is a random error term. In the regression results of model (15), the coefficient of the quadratic term Wage is the focus of our attention. If the coefficient of the quadratic term is positive, it indicates that there is a U-shaped relationship between the degree of foreign capital control over producer services and the wage level of manufacturing industry, that is, there is an inverted U-shaped relationship between the opening of producer services and the wage growth of manufacturing industry.

### 4.3 Variable description

#### 4.3.1 Wage level of manufacturing industry (Wage)

This paper refers to the practice of Hou and Sun (2019) [47] and uses the annual per capita wage of enterprises in the industry after excluding domestic price CPI to express the actual wage level of the industry. To eliminate the influence of heteroscedasticity and increase the stationarity of the data, this variable is logarithmically transformed.

#### 4.3.2 Degree of foreign capital control in producer services (Msfdi)

There are three main ways to measure the openness of producer services to the outside world: the first is "promised openness". Using the data provided by the WTO (World Trade Organization) database, the openness of the service industry is measured by examining the proportion of the number of sectors promised to open in a country’s GATS (agreement on trade in services) to the total number of sectors [48]. The second is "policy openness", which is weighted by the Service Trade Restriction Index (STRI) published by the World Bank. The third is "actual openness", which is usually measured by the degree of dependence on foreign trade or foreign investment [49, 50] to reflect the actual effect of various countries’ service industry opening measures. This paper constructs the opening index of producer services based on China’s foreign investment participation and opening policy in the service industry, which avoids the endogenous problem of taking the performance of the service industry market as a measure of the opening level to a certain extent.

Considering the endogeneity problem and data availability, this paper constructs the foreign capital opening index from the perspective of foreign capital control. Specifically, the catalogue for the Guidance of Foreign Investment Industries (Referred to as “Catalogue”) specifies in detail the items that are prohibited, restricted, and encouraged for foreign investment in each subindustry of the service industry. The industries that are not listed are deemed to be allowed for foreign investment, and each item is manually connected with the service industry classification of the quartile of the national economy in 2002. Second, the degree of foreign capital control (*Sfdi*) of each service industry is assigned, and the service industries with prohibited, restricted, permitted, and encouraged items are assigned 1, 0.5, 0.25 and 0, respectively. The smaller the value of *Sfdi is*, the lower the degree of foreign capital control of the industry and the higher the degree of openness. Finally, using the input‒output coefficient (*W*_*sj*_) of the service industry to the manufacturing industry in the input‒output table of 122 departments in China in 2002 as the weight, the productive service restriction index of the manufacturing industry is constructed.


$$Msfdi_{jt}=\sum_{s}Sfdi_{st}\times w_{sj}$$

Among them, subscripts s and *j* represent the productive service industry and each subindustry of the manufacturing industry, respectively. *Msfdi*_*jt*_ is the foreign capital restriction degree of the upstream service industry of manufacturing industry *j*, *Sfdi*_*st*_ is the foreign capital control degree of service industry *s* in year *t*, and *W*_*sj*_ is the total investment proportion of service industry *s* in manufacturing industry *j*. The smaller *Msfdi*_*jt*_ is, the lower the degree of regulation of the upstream service industry of the manufacturing industry and the higher the degree of openness.

#### 4.3.3 Other variables

Labour productivity in the manufacturing industry (Productivity). The ratio of the added value of each subindustry of the manufacturing industry to the employees in the industry is taken as the natural logarithm.

Labour income share of manufacturing industry (Share). The share of labour income is expressed by the ratio of the remuneration of workers in the manufacturing industry to the added value of the manufacturing industry [44]. The specific components of industrial added value include depreciation of fixed assets, labour remuneration, net production tax and operating surplus [51].

Capital intensity (*Capital*) of the industry is measured by the ratio of the total fixed assets of the manufacturing industry to the total number of employees. Compared with labour-intensive enterprises, the per capita wage of workers in capital-intensive enterprises increases faster, and the wage level is higher [52]. Industry size (*Size*) is expressed as the logarithm of the total assets of enterprises in the industry. For listed companies, uneconomical scale or excessive enterprise size will lead to a decline in the bargaining power of workers and thus inhibit wage growth [53]. The degree of industry competition (*HHI*) is expressed by the Herfindahl index, which is obtained by the sum of the squares of the market share of each enterprise in the industry. The higher the *HHI* value is, the higher the industrial concentration and the higher the degree of industry monopoly. When an enterprise is in a highly monopolistic industry, the stronger the ability of the enterprise to obtain income, the greater the possibility of workers obtaining high wages. Generally, the worse the solvency of an enterprise is, the worse the capital liquidity and financial condition of the enterprise, which affect the salary growth of employees. Therefore, the average equity ratio (*Equity*) is included in the regression model. The operating status of enterprises is linked to the profit distribution of enterprises, which directly affects the distribution of labour income of enterprises. Therefore, the return on net assets (*ROE*) is taken as the performance of enterprises in the control variable.

## 5 Results

### 5.1 Benchmark regression results

**Fig 2:**
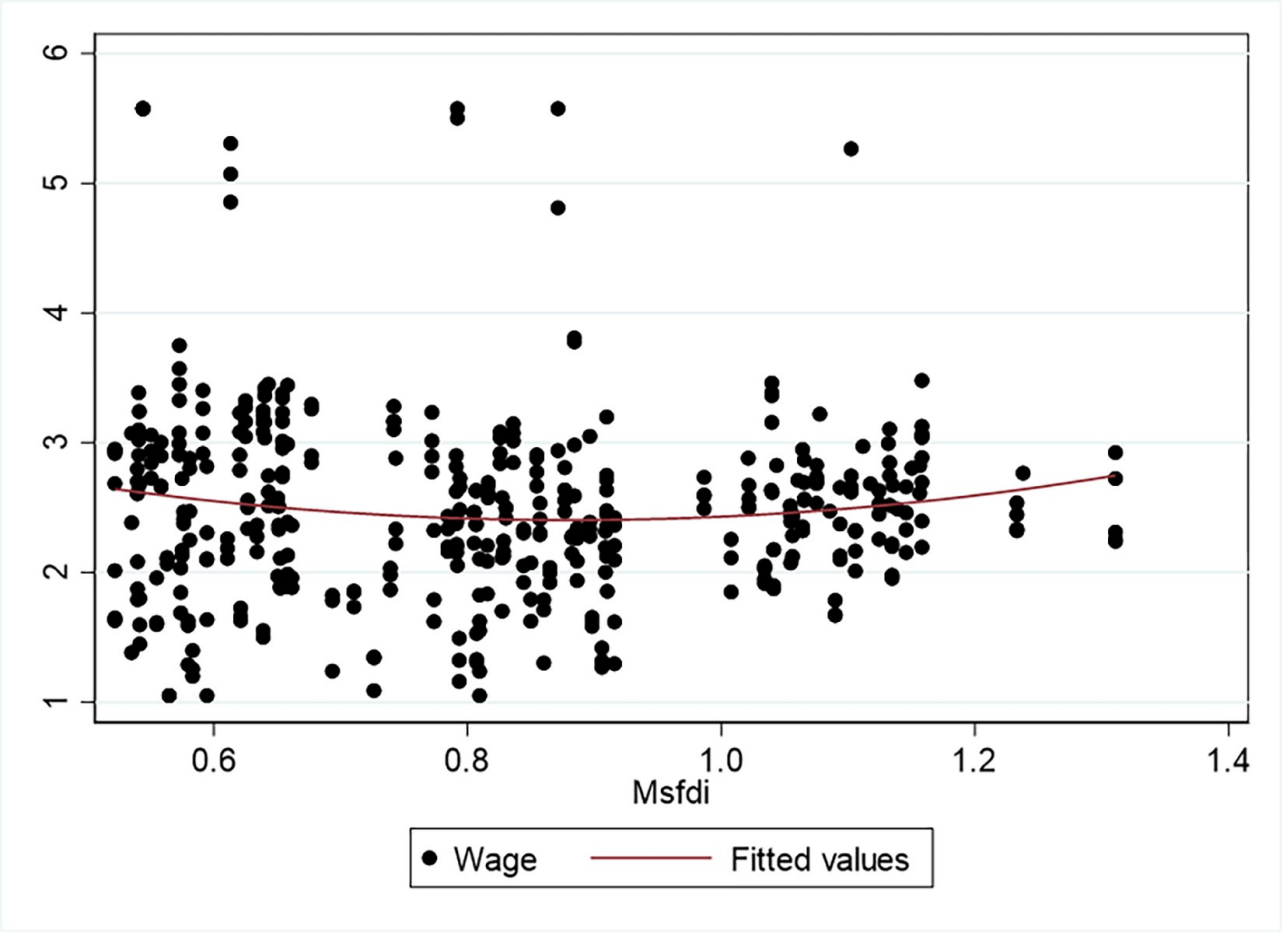
Fitting graph of the logarithm of the wage level of the manufacturing industry with the opening of producer services.

Fig 2 shows the scatter plot and fitting plot with the constraint degree of the producer service industry as the independent variable and the logarithm of the wage level of the manufacturing industry as the dependent variable. Although the scatter plot and fitting plot enable us to intuitively draw the relationship between the two variables, a more accurate relationship can only be obtained by eliminating the interference of other variables.

Columns (1) and (2) of Table 2 report the regression results of the linear relationship between the opening of producer services and the wage growth of downstream manufacturing industries. Column (1) does not include control variables, and Column (2) includes five control variables, including asset intensity, industry scale, industry competitiveness, property rights ratio and return on net assets, and controls the fixed effects of years and industries. The regression results show that the estimated coefficients of foreign capital regulation in producer services are all negative but lack statistical significance, which means that the impact of foreign capital regulation in producer services on manufacturing wages is not obvious. We can further investigate whether there is a nonlinear relationship between foreign capital regulation in producer services and manufacturing wages.

**Table 2:** Benchmark regression results of the impact of the opening of producer services on manufacturing wages.

	Wage			
	(1)	(2)	(3)	(4)
Msfdi	-0.825 (-1.29)	-0.792 (-1.33)	-4.386** (-2.19)	-3.626** (-2.16)
Msfdi^2^			2.187** (2.15)	1.741** (2.07)
Capital		0.152* (1.87)		0.159* (1.86)
Size		-0.344*** (-3.23)		-0.343*** (-3.26)
HHI		3.078** (2.23)		2.803** (2.12)
Equity		-0.003*** (-2.56)		-0.003** (-2.23)
ROE		3.449*** (3.50)		3.432*** (3.51)
Year/Idustry FE	Yes	Yes	Yes	Yes
Constant	2.198*** (3.94)	2.849*** (4.16)	3.570*** (3.52)	3.969*** (3.87)
Hausman test	10.32 (0.001)	39.96 (0.000)	9.04 (0.011)	38.96 (0.000)
R^2^	0.469	0.524	0.477	0.529
Observations	418	418	418	418

Note: T statistics are in parentheses

*, * *, * * * are significant at 10%, 5% and 1% respectively, and the result of the Hausman test has a p value in parentheses.

Columns (3) and (4) of Table 2 report the results of nonlinear regression between the opening of producer services and the wage growth of downstream manufacturing industries. Column (3) does not include control variables, and Column (4) includes control variables, time fixed effects and industry fixed effects. The regression results show that the estimated coefficient of the primary term and the estimated coefficient of the secondary term of foreign capital regulation in producer services are negative and positive, respectively, and both are significant at a certain level, indicating that there is a significant positive U-shaped relationship between foreign capital regulation in producer services and manufacturing wages and an inverse U-shaped relationship between the opening of producer services and manufacturing wages, which verifies Hypothesis 1. Specifically, when the openness of producer services is lower than a certain threshold, the openness of producer services will improve the wage level of downstream manufacturing industries. When the openness of producer services is higher than a certain threshold, the openness of producer services will inhibit the growth of wages in the downstream manufacturing industry. Based on the estimated coefficients of the primary and secondary terms of foreign investment control in producer services in Columns (3) and (4), the thresholds of foreign investment control in producer services are calculated to be 1.0027 and 1.0432, respectively. At present, the average value of foreign capital regulation in producer services is 0.9022, and the opening of producer services is still in the stage of promoting the wage growth of the manufacturing industry. The Hausman test results show that the null hypothesis of random effects is rejected, so a fixed effects model is established.

### 5.2 Robustness test

#### 5.2.1 Reinspection of enterprise level data

The previous text mainly used data at the manufacturing industry level for empirical analysis. Here, the micro level data of manufacturing A-share listed companies from 1999 to 2020 are used for re-examination. The results in Columns (1) and (2) of Table 3 show that the primary term coefficient of foreign capital regulation in producer services is significantly negative, and the secondary term coefficient is significantly positive at the 5% level. Therefore, the basic conclusions of this paper have not changed greatly due to the different samples.

**Table 3:** Robustness test results.

	Reinspection of enterprise level data		Replace the interpreted variable	Replace explanatory variables	Endogenous problem	Consideration of lag effect
	(1)	(2)	(3)	(4)	(5)	(6)
**Msfdi**	-1.701* (-1.77)	-1.963** (-2.22)	-1.925*** (-3.99)	-2.338*** (-6.18)	-35.563 (0.278)	-4.058** (-2.41)
**Msfdi** ^**2**^	0.734** (2.22)	0.795** (2.52)	0.776*** (3.09)	1.157*** (4.28)	4.966* (0.080)	1.885** (2.30)
**Control variable**	No	Yes	Yes	Yes	Yes	Yes
**Year/ Industry FE**	Yes	Yes	Yes	Yes	Yes	Yes
**Constant**	4.125*** (5.79)	3.140*** (4.77)	12.046*** (43.27)	9.728*** (47.74)	31.645 (0.276)	4.691*** (3.99)
**R** ^**2**^	0.189	0.257	0.991	0.967	0.142	0.516
**Observation**	22107	22107	418	418	11663	399
**LM statistic**					250.119	
**F statistic**					100.308	

Note: Except for the p value in the brackets of Column (5), all the other columns are t statistics, and

*, * *, * * * indicate that they are significant at the levels of 10%, 5% and 1%, respectively.

#### 5.2.2 Replacement core variable

Replace the interpreted variable. In the previous section, the actual wage level is constructed by using the sum of the employee compensation payable and the welfare payable after excluding the domestic price CPI in the manufacturing industry. Here, the per capita "cash paid to and for employees" after excluding the domestic price CPI in the manufacturing industry is used to measure the per capita actual wage level in each branch of the manufacturing industry. After replacing the explained variables, the basic conclusions of this paper have not changed significantly.

Replace explanatory variables. In the previous article, China’s input‒output table in 2002 was mainly used to construct the indicators of foreign investment opening in producer services. Here, China’s input‒output tables in 2002, 2007, 2012 and 2017 were used to calculate the weight to construct the degree of foreign investment control in producer services. After replacing the explanatory variables, the basic conclusions of this paper have not changed significantly.

#### 5.2.3 Endogeneity problem

Since the opening of producer services and the profit distribution behaviour of manufacturing enterprises may be influenced by some unobserved factors at the same time, the India service trade restriction index (STRI) released by the OECD is used here to construct the instrumental variable [3]. China and India have certain similarities in foreign investment policies, and the wage payment behaviour of domestic manufacturing enterprises will not have an impact on India’s producer services trade control policy, which better meets the correlation and exogenous conditions of instrumental variables. Due to the limitation of sample size, endogeneity problems are tested using enterprise-level data. After considering the possible endogeneity problems, the conclusion of this paper has not changed much.

#### 5.2.4 Consideration of lag effect

Economic theory shows that the influence between economic variables needs a lag period to play a role. The core explanatory variables and other control variables are all delayed by one period, and the regression is carried out by replacing them with the econometric model set up above. Column (6) in Table 3 reports the estimated results of producer service opening and core explanatory variables, and the coefficients are significantly positive at the 5% level, which is consistent with the previous results. The above analysis shows that the lag between variables has no substantial impact on the above conclusions, and the conclusions of this study are robust to a certain extent.

## 6 Mechanism test and heterogeneity analysis

### 6.1 Mechanism test

To test the two channels of labour productivity and labour income share that the opening of producer services affects the wage level of the manufacturing industry, model (16) is constructed to test the two mechanisms.

$$M_{jt}=\alpha_{0}+\alpha_{1}Msfdi_{jt}+\alpha_{2}Msfdi_{jt}^{2}+\varphi X_{jt}+\mu_{j}+\mu_{t}+\varepsilon_{jt}$$ (16)

where *M*_*jt*_ represents the labour productivity (*Productivity*_*jt*_) and labour income share (*Share*_*jt*_) of the manufacturing industry in the t-th year, respectively. According to the mechanism test results in Table 4, it can be found that the first-order coefficient of foreign capital regulation in producer services on labour productivity in the manufacturing industry is negative, the coefficient of the quadratic term is positive, and both are significant at a certain level, which indicates that the opening of producer services has an inverted U-shaped impact on the labour productivity of the manufacturing industry, which proves Hypothesis 2. According to the primary and secondary coefficients, the threshold of foreign capital regulation in producer services is calculated to be 1.343. The effect of opening producer services on labour productivity in the manufacturing industry is still in the promotion stage. Furthermore, after adding labour productivity into the benchmark model, the estimated coefficient of labour productivity is significantly positive, which confirms the impact of the opening of producer services on manufacturing wages from the perspective of labour productivity.

**Table 4:** Mechanism test of the impact of the opening of producer services on the wage growth of the downstream manufacturing industry.

	Productivity (1)	Share (2)
**Msfdi**	-1.305** (-2.44)	-3.654*** (-2.65)
**Msfdi** ^**2**^	0.486* (1.78)	2.676*** (3.36)
**Capital**	0.099*** (2.96)	-0.129 (-1.45)
**Lnsize**	0.816*** (16.08)	0.971*** (8.25)
**HHI**	0.272 (0.65)	1.356 (1.56)
**Equity**	-0.364 (-0.50)	-1.664 (-1.48)
**ROE**	0.455 (1.10)	-1.747** (-2.50)
**Year/Industry FE**	Yes	Yes
**Constant**	17.241*** (40.77)	-3.562*** (-3.98)
**R** ^**2**^	0.986	0.782
**Observation**	418	418

Note: T statistics are in parentheses

*, * *, * * * are significant at 10%, 5% and 1% respectively.

From the estimation results in Column (2) of Table 4, it can be seen that the first term coefficient of foreign capital control in producer services is negative, the coefficient of the quadratic term is positive, and both are significant at the level of 1%, which indicates that the opening of producer services has an inverted U-shaped impact on the labour income share of the manufacturing industry, which proves Hypothesis 3. This is consistent with the previous analysis. When labour and service inputs are complementary, the opening of producer services increases the demand for highly skilled workers, and higher wage bargaining power significantly increases the share of labour income in the manufacturing industry. However, when labour and productive service inputs become substitutes, the labour income distribution effect of the opening of producer services will change from positive to negative. According to the primary and secondary coefficients, the threshold value of foreign capital regulation in producer services is calculated to be 0.683. At present, the average value of foreign capital regulation in producer services is higher than the threshold value. The opening of producer services is still in the stage of inhibiting the increase in labour income share in the manufacturing industry. Furthermore, after the labour income share is added to the benchmark model, its estimation coefficient is significantly positive, which confirms the impact of the opening of producer services on manufacturing wages from the perspective of the labour income share.

### 6.2 Heterogeneity analysis

#### 6.2.1 Industry factor density

In this paper, Stehrer et al’s method (2012) [54] and other classification methods of the manufacturing industry are used, which are divided into three categories: labour-intensive manufacturing industry, capital-intensive manufacturing industry and technology-intensive manufacturing industry. From the heterogeneity test results in Table 5, it can be seen that the opening of producer services can significantly improve the wage level of downstream capital and technology intensive manufacturing industries, but the linear and nonlinear relationship with the wage level of labour-intensive manufacturing industries lacks statistical significance, which indicates that the more capital and technology intensive the manufacturing industry, the more significant the effect of the opening of upstream producer services on the wage of manufacturing industry, This coincides with Acemoglu’s (2010) [55] and others’ view of skill biased technological progress. For the labour-intensive manufacturing industry, its production factors are low-skilled labour and physical capital, and the complete consumption coefficient of the producer service industry is small. The producer service industry is not a necessary intermediate input for the labour-intensive manufacturing industry, so it plays a small role in the wage growth of the manufacturing industry. Capital and technology-intensive manufacturing industries have higher requirements for knowledge and information sharing and high-quality talent in agglomeration economies and are greatly affected by producer services.

**Table 5:** Heterogeneity test results.

	Wage			
	Labor intensive industries (1)	Capital and technology intensive industries (2)	Highly competitive industries (3)	Low competitive industries (4)
**Msfdi**	0.472 (0.32)	-8.087** (-2.38)	-11.430*** (-2.78)	-10.294*** (-3.00)
**Msfdi** ^**2**^	-0.155 (-0.19)	-0.273 (-0.21)	6.286*** (2.79)	-1.207 (-1.18)
**Control**	Yes	Yes	Yes	Yes
**Year/ Industry FE**	Yes	Yes	Yes	Yes
**Constant**	2.489** (2.44)	7.452*** (3.44)	8.823*** (4.11)	7.734*** (3.54)
**R** ^**2**^	0.857	0.510	0.537	0.643
**Observation**	110	308	220	198

Note: T statistics are in parentheses

*, * *, * * * are significant at 10%, 5% and 1% respectively.

#### 6.2.2 Industry competition

This paper uses Zhang (2020) [56] for reference to divide the manufacturing industry into highly competitive industries and low competitive industries. The first-order coefficient of foreign capital regulation in producer services on the wage level of the highly competitive manufacturing industry is significantly negative, and the second-order coefficient is significantly positive, indicating that the opening of producer services presents an inverse U-shaped relationship of first increasing and then inhibiting the wage growth of the highly competitive manufacturing industry. However, for the manufacturing industry with a high monopoly, the first-term coefficient of foreign capital control in producer services is significantly negative, and the quadratic coefficient is negative but lacks statistical significance, which indicates that the positive correlation between the opening of producer services and wage growth in the manufacturing industry with a high monopoly is established. The market share of the domestic low-competitive manufacturing industry is more stable, and it is not easily affected by the opening of the service industry. The cost saving effect enables low-competitive manufacturing enterprises to quickly weigh costs and benefits and make the optimal production decision to promote corporate profits, which is more conducive to wage growth.

## 7 Discussion

### 7.1 Results and discussion

In this paper, we studied the impact mechanism and heterogeneity of the opening of producer services on wages in China’s manufacturing labour market. We obtained three main results. First, according to the wage growth model, the wage growth rate is affected by the growth rate of labour productivity and the growth rate of labour income share. It can be seen from the data in Table 4 that when the substitution of domestic products or services for foreign products or services is weak, local manufacturing enterprises can promote the labour productivity of downstream manufacturing industries by further deepening the professional division of labour, improving the technical level and optimizing the supply and allocation of factors in the "division of labour effect", "technology spillover effect" and "resource allocation effect" brought about by the opening of producer services. The positive effect of the opening of producer services is greater than the negative effect brought about by the "competition effect". On the whole, it promotes the growth of labour productivity in the manufacturing industry and is conducive to the growth of wage levels, this conclusion is basically consistent with Gu and Dai [25]. However, with the continuous improvement of the level of opening to the outside world and the gradual shift of the economy to high-quality development, the position and brand value of the manufacturing industry in the global value chain have been greatly improved [57], the substitutability between domestic and foreign products has gradually increased [58], and the substitutability between nonservice input and service input has also been continuously enhanced [59], that is, σ>1. The "competitive effect" of the opening of producer services through the "Introduction" is greater than the positive effect, which has a negative impact on the labour productivity of the manufacturing industry [37] and inhibits the growth of the wage level of the manufacturing industry.

Second, when the productive service input and labour are complementary, local manufacturing enterprises make use of the "direct effect" generated by the opening of the producer service industry to obtain biased technological progress and improve the remuneration of workers. The positive effect on the labour income share is greater than the negative effect brought by the "indirect effect", which promotes the wage growth of the manufacturing industry. As the economy turns from an "industrial economy" to a "service economy", the backwards brand value and insufficient technical capacity of the traditional manufacturing industry have been gradually improved, the investment in capital- and technology-intensive services has also been further increased, and the productive service factors and labour factors have been replaced (ε>1). Moreover, since the manufacturing industry in developing countries is located in a relatively low position in the global value chain and the space for product quality differentiation is small, it cannot be converted into wage earnings. The labour income distribution effect of the opening of producer services will change from positive to negative [44], thus inhibiting wage growth in the manufacturing industry.

The results in Table 4 show that the opening of producer services and the labour productivity and labour income share of the manufacturing industry show a nonlinear inverse U-shaped relationship, and labour productivity and labour income share are two important channels through which the opening of producer services improves the wage growth of the manufacturing industry. This result explains why the quadratic coefficient of producer services restriction on manufacturing wage growth is positive (Table 2); that is, the opening of producer services presents an inverse U-shaped relationship with manufacturing wage growth.

Finally, we find that there is industry heterogeneity in the effect of the opening of producer services on manufacturing wage growth. The empirical results in Table 5 show that the wages of capital and technology intensive and low competitive manufacturing industries are relatively strongly promoted by the openness of producer services. Generally, there are a large number of enterprises in highly competitive industries. Existing studies have focused on the impact of opening to the outside world on wage inequality, arguing that opening up expands the income gap or reduces poverty, which coincides with our conclusion and confirms the heterogeneity analysis results. However, as most of the highly competitive industries are light industrial sectors of the manufacturing industry, with the continuous improvement of the opening level of upstream producer services, their position in vertical specialization is easily marginalized, and they are at the bottom of the smile curve in the division of labour in the global value chain [48]. They have not obtained higher added value and global value chain status, and the negotiation ability of labour factors has also declined, affecting the improvement of labour income share. On the other hand, when the manufacturing industry is more capital intensive and the technology level is higher, enterprises will have more advantages in realizing industrial upgrading by taking advantage of the "ripple effect" brought about by the opening of producer services. The opening of upstream producer services drives the technological progress of the downstream manufacturing industry to accelerate the depreciation of material capital, while human capital shows stronger game power in the game with material capital [60]. Knowledge and technology are also injected into all links of product production, and the opening of producer services has a stronger positive effect on wage growth in the manufacturing industry. Compared with the existing studies, in terms of research topic, this paper explores the impact of the opening of producer services on the wage growth of manufacturing industry based on the perspective of vertical correlation between industries. In terms of theoretical mechanism, productive services and production input factors are integrated into the wage growth model of manufacturing enterprises, and a theoretical framework is built that the opening of producer services affects the wage growth of manufacturing industry through labor productivity and labor income share. In the empirical analysis, the index of producer service opening which can avoid endogeneity is constructed, and the difference of producer service opening on wage growth in different manufacturing industries is discussed.

### 7.2 Conclusions and policy implications

Under the background of expanding the opening of producer services and slowing down the wage growth of the manufacturing industry, this paper brings the opening of producer services and the wage growth of the manufacturing industry into a unified analysis framework at the micro level and constructs a theoretical model of the impact of the opening of producer services on the wage growth of the downstream manufacturing industry from the perspectives of labour productivity and labour income share. Then, we use manufacturing industry-level data from 1999 to 2020 for testing. The results show that the opening of producer services has an inverted U-shaped effect on the wage growth of the downstream manufacturing industry, which is first increased and then suppressed. This conclusion is still valid after a series of robustness tests, such as endogenous tests. Further mechanism tests have confirmed that the opening of producer services has an inverted U-shaped relationship with the labour productivity and labour income share of the manufacturing industry, which is an important channel to affect the wage growth of the manufacturing industry. This paper also conducts a heterogeneity analysis from the two aspects of industry factor density and industry competition. The analysis results show that the opening of producer services has significantly promoted the wage growth of capital and technology intensive and low competitive manufacturing industries. The “promotion effect” brought about by the opening of the producer services industry will promote the international division of labour and industrial transformation of the manufacturing industry, promote the sustainable growth of the labour productivity and labour income share of the manufacturing industry, drive the rise of the wage level of the manufacturing industry, further release the domestic consumption potential, and coordinate the tension between fairness and efficiency in the initial distribution.

Our results have some implications for China’s manufacturing industry to better enjoy the dividend of the opening of producer services. First, the formulation of policies should focus on promoting the two-way opening of producer services and building a "Made in China" brand. We should pay attention to promoting high-standard opening up, focus on absorbing and introducing productive service enterprises with high relevance and high spillover, and improve the input quality of local producer service elements through digestion, absorption and re-innovation. Governments should also continue to encourage Chinese enterprises to "go global", release the technology spillover effect of foreign enterprises by learning from and imitating foreign high-tech enterprises, promote technological progress in domestic service fields, inject high-quality productive service elements into manufacturing enterprises, and enhance the value of independent brands.

Second, we should deepen the leading role of producer services in the transformation and upgrading of the manufacturing industry and drive the realization of the "long chain" of China’s manufacturing industry chain. The effect of the opening of producer services on the increase in real wages in capital- and technology-intensive manufacturing is significantly greater than that in labour-intensive manufacturing. Therefore, low value-added enterprises should be encouraged to undertake service outsourcing businesses such as logistics distribution, distribution and warehousing, testing and maintenance, R&D and design, and extend the processing trade industry chain. We should make full use of the opportunity of the opening of producer services to learn from international cutting-edge marketing services, information collection, R&D innovation and other capabilities, promote its transformation to a manufacturing industry with higher technology and higher added value, break the shackles of "low-end locking", and drive the improvement of the actual wage level of the manufacturing industry.

Third, the Chinese government should actively take measures to promote the free flow of labour factors, optimize the allocation of resources, and promote the sustainable growth of labour productivity and labour income share to achieve the sustainable growth of wages and overcome the "middle-income trap". Local governments should actively reform the registered residence system and establish a unified labour market so that labour can move freely between urban and rural areas and regions and industries and employment levels can be consistent. We should promote the reform of income distribution and build a harmonious labour capital relationship and especially improve the collective bargaining system and strengthen the role of trade unions in safeguarding workers’ rights and interests. The low technology level and added value of labour-intensive industries based on a cheap labour force lead to low profit margins of enterprises and restrict the growth of workers’ wages. Therefore, we should accelerate the transformation of the economic development mode and realize the upgrading of industrial structure as soon as possible, which will be conducive to the improvement of workers’ income.

Fourth, we need to accelerate the establishment of competitive advantages in human resources and guide the transformation from a "demographic dividend" to a "talent dividend". The opening of producer services requires the matching of highly skilled labour, the establishment of a comprehensive talent training system for modern services and manufacturing, the improvement of labour quality and mobility of labour factors, and the promotion of rational allocation of labour resources to ensure the effective operation of the opening of producer services through the mechanism of the labour structure effect.

### 7.3 Limitations and future research

Our research is not unlimited. Considering the wide range of sample years and the availability of data, we use the data at the level of Chinese manufacturing listed enterprises to obtain the data at the level of the manufacturing industry through summary and processing. We do not include the data of unlisted companies in the empirical scope. However, as Du (2009) pointed out [61], listed companies, as the main force and leader in promoting the sound and rapid development of China’s economy, have a clear response to macroeconomic policies, which is helpful for analysing the macro impact. Therefore, it is reasonable for this paper to choose listed companies as research samples. It should be noted that our research focuses on the impact of the openness of producer services on the manufacturing industry. Since producer services are more open than life services, service trade is also dominated by producer services. When measuring the openness of producer services, we also use the openness of services [62].

The results of this paper provide some ideas for China to fully release the dividend of productive services and promote the growth of manufacturing wages, which may not be applicable to other countries. Is there any heterogeneity in the impact of producer services opening on manufacturing wage growth in different countries? In the context of the current digital economy, can digital technology promote economic restructuring and help China overcome the "middle-income trap"? How can digital technology be used to better realize the supervision, risk prevention and control of the cross-border service trade? These problems need to be solved in future research.

## Supporting information

S1 Data (ZIP)
